# Different Resistance to UV-B Radiation of Extracellular Polymeric Substances of Two Cyanobacteria from Contrasting Habitats

**DOI:** 10.3389/fmicb.2016.01208

**Published:** 2016-08-22

**Authors:** Wenjuan Song, Chenxi Zhao, Daoyong Zhang, Shuyong Mu, Xiangliang Pan

**Affiliations:** ^1^Xinjiang Key Laboratory of Environmental Pollution and Bioremediation, Xinjiang Institute of Ecology and Geography – Chinese Academy of Sciences, UrumqiChina; ^2^Xinjiang Academy of Environmental Protection Sciences, UrumqiChina; ^3^State Key Laboratory of Environmental Geochemistry, Institute of Geochemistry – Chinese Academy of Sciences, GuiyangChina

**Keywords:** chlorophyll fluorescence, decomposition, EEM fluorescence spectroscopy, EPS, photosynthesis

## Abstract

The effects of UV-B radiation (UVBR) on photosynthetic activity (Fv/Fm) of aquatic *Synechocystis* sp. and desert *Chroococcus minutus* and effects on composition and fluorescence property of extracellular polymeric substances (EPSs) from *Synechocystis* sp. and *C. minutus* were comparatively investigated. The desert cyanobacterium species *C. minutus* showed higher tolerance of PSII activity (Fv/Fm) to UVBR than the aquatic *Synechocystis* sp., and the inhibited PSII activity of *C. minutus* could be fully recovered while that of *Synechocystis* sp. could be partly recovered. UVBR had significant effect on the yield and biochemical composition of EPS of both species. Protein-like and humic acid-like substances were detected in EPS from *Synechocystis* sp., and protein-like and phenol-like fluorescent compounds were detected in EPS from *C. minutus.* Proteins in EPS of desert and aquatic species were significantly decomposed under UVBR, and the latter was more easily decomposed. The polysaccharides were much more resistant to UVBR than the proteins for both species. Polysaccharides of *Synechocystis* sp. was degraded slightly but those of *C. minutus* was little decomposed. The higher tolerance to UVBR of the desert cyanobacterium can be attributed to the higher resistance of its EPS to photodegradation induced by UVBR in comparison with the aquatic species.

## Introduction

Ultraviolet-B radiation (UVBR) at 280–315 nm is one of the major detrimental environmental factors for photosynthetic organisms in arid regions (Bowker et al., 2002). Depletion of the ozone layer leads to increasing levels of UVBR reaching the earth’s surface (Madronich et al., 1995).

Cyanobacteria are important photosynthetic microorganisms that live widely in aquatic and terrestrial environments. Exposure to UVBR can be potentially harmful for cyanobacteria in case the provoked damage exceeds the capacity of defense and repair mechanism (Sutherland, 2001). Some studies have shown that UVBR could induce physiological changes, including inhibition and even damage to photosystem, and enhance the content of reactive oxygen species (ROS) in cyanobacterial cell (Kumar et al., 2004). The high level of ROS is potentially harmful to the normal metabolism and results in oxidative injury such as protein degradation and DNA damage (He and Häder, 2002). Cyanobacteria have developed many mechanisms to against UVBR (Sinha et al., 1998). For example, cyanobacteria can synthesize UV-B absorbing compounds to scavenge ROS, or escape from UV-B damage by migration (Quesada and Vincent, 1997).

As ubiquitous species in aquatic and terrestrial habitats, most cyanobacteria are capable of secreting large quantities of extracellular polymeric substances (EPSs), which are mainly composed of polysaccharides, proteins, humic substances, and nucleic acids (Liu and Fang, 2002; Zhu et al., 2012). EPS has important cellular functions, including accumulation of nutrients, cell motility formation of barrier for toxins, attachment to surfaces and contribution for biofilms (Nichols et al., 2005; Ozturk et al., 2009; Cosenza et al., 2013). Synthesis of EPS in cyanobacteria leads to a better survival capacity compared to many other phototrophic microorganisms under UV radiation (Helm et al., 2000). EPS of cyanobacteria form a buffer zone between living cell and environment to counteract the adverse effects of UV radiation. The free radicals-scavenging ability of EPS plays a major role in the tolerance of bacteria to UV radiation (Wang et al., 2007). However, the composition and structure of EPS vary with cyanobacteria species and environmental conditions (Stal and Krumbein, 1985). Some studies showed that the monosaccharides component, molecular weight, and proteins content of EPS may affect their activities to counteract with UVBR (Calazans et al., 2000).

As the most important protective barrier, little data on the response of photosynthetic activity of cyanobacteria and the protective function of their EPS to UVBR are available. In the present study, the freshwater *Synechocystis* sp. and the desert *Chroococcus minutus* are chosen as the model cyanobacteria to study the decomposability of EPS and its effect on growth and photosynthetical activity of cyanobacteria under UVBR.

## Materials and Methods

### Organisms and Culture Conditions

One freshwater *Synechocystis* sp. and one desert *C. minutus* were selected in this study. The *Synechocystis* sp. (FACHB-898) was obtained from the Institute of Hydrobiology – Chinese Academy of Sciences. The *C. minutus* was isolated and purified from desert soil. Cyanobacterial cells were grown in BG-11 medium (Stanier et al., 1971) at 30°C and an irradiance of 55 μmol photons/m^2^⋅s PAR with a 12-h light: dark photoperiod.

### EPS Extraction

Extracellular polymeric substance were extracted by high-speed centrifugation (Zhang et al., 2010; Pan et al., 2012). The culture was centrifuged at 5500 rpm for 10 min at 4°C. The cyanobacterial cells were re-suspended in deionized water. Then the cell suspension was centrifuged at 12300 rpm for 20 min at 4°C. The supernatant was filtrated through 0.45 μm acetate cellulose membrane and purified with dialysis membrane (3500 Da) for 24 h at 4°C.

### UV-B Treatments

The glass plates containing the cultures and the glass plates containing only EPS were placed in a sterilized cabinet at room temperature under both photosynthetically active radiation (PAR) and UVBR. The radiation of 55 μmol photons/m^2^⋅s PAR was supplied by white fluorescent light, and the UVBR of 610 μW/cm^2^ (6.1 J/m^2^⋅s) was supplied by a ultraviolet radiation lamp (EB-160C/FE, USA) with the peak emission at 312 nm. The UV-B lamp was turned on 30 min prior to use to allow for stabilization of the UV output. The UV-B exposure time of cyanobacteria cells is 0, 1, 2, 5, 10, 15, and 20 min. The exposure time of EPS was 0, 1, 2, 3, 6, 9, and 12 h. In order to prevent the evaporation of solutions during radiation, cellulose acetate membranes were covered on the plates. After exposure to UVBR, the plates containing cyanobacteria cells were placed in artificial climate box at room temperature under an irradiance of 55 μmol photons/m^2^⋅s PAR with a 12-h light: dark photoperiod for recovery.

### Determination of Chlorophyll a Fluorescence and Biomass

The chlorophyll *a* fluorescence was measured with a double-modulation fluorometer (FL3500, PSI, Brno, Czech). Cyanobacteria cells were dark-adapted for 5 min before measuring the fluorescence parameter Fv/Fm. Fv/Fm is the maximum photochemical efficiency of photosystem II and it is a most commonly used photosynthetic function parameter for photosynthetic microorganisms (Zhou et al., 2006).

The living cell biomass was represented by the optical density of cell suspension at 680 nm. The value of OD_680_ was measured with an ultraviolet-visible spectrophotometer (UNIC2800, Shanghai, China).

### Determination of Biochemical Composition of EPS

Content of polysaccharides was measured by phenol sulfuric acid method, using glucose as the standard (Dubois et al., 1956). The content of proteins was measured by Bradford’ method, with bovine serum albumin as the standard (Bradford, 1976). Content of total organic carbon (TOC) in EPS solution was measured with a TOC analyzer (TOC-VCTH, Shimadzu, Japan). The molecular weight distribution was measured by size exclusion high performance liquid chromatography (HPLC-SEC; HPLC, Hitachi, Japan). EPS were subjected to gel permeation chromatography on column with Superdex TM 20010/300GL (9 mM NaCl+0.9 mM Na_2_HPO_4_, flow rate 0.4 ml/min). The standard substance with molecular weight in the range from 14.4 to 94 kDa from Polymer Standards Service was used to calibrate the retention time. The results were expressed as the relative (sum of all pigment classes by mass = 100%) amounts of molecular weight.

### EEM Fluorescence Spectroscopy

The excitation emission matrix (EEM) spectra of EPS were measured by a fluorescence spectrophotometer (F-7000, Hitachi, Japan) equipped with 1.0 cm quartz cell and a thermostat bath (Pan et al., 2010, 2012; Song et al., 2010). EEM spectra were collected with subsequent scanning emission wavelength from 200 to 550 nm at 2 nm increments and excitation wavelength from 200 to 400 nm at 5 nm increments, with an excitation/emission slit width of 5.0 nm and a scanning speed of 1200 nm/min. The fluorometer’s response to a Milli-Q water blank solution was subtracted from the fluorescence spectra of EPS. Three-dimension EEM data were processed using the software SigmaPlot 10.0 (Systat, USA).

### Staining of EPS

Extracellular polymeric substance of cyanobacteria cells were observed by alcian blue staining method (Wardi and Allen, 1972), the cells were washed six times with sterilized water and then fixed on a slide by adding a few drops of 4% formaldehyde for 12 h. Cells on slides were then stained with alcian blue (10% w/v in 0.5 M acetic acid). After 12 h of staining the slides were rinsed with distilled water. The cells and stained EPS were observed using a light microscope (XSZ-HYZ, COIC, China) equipped with a camera.

## Results and Discussion

### Effect of UVBR on PSII Activity

**FIGURE 1:**
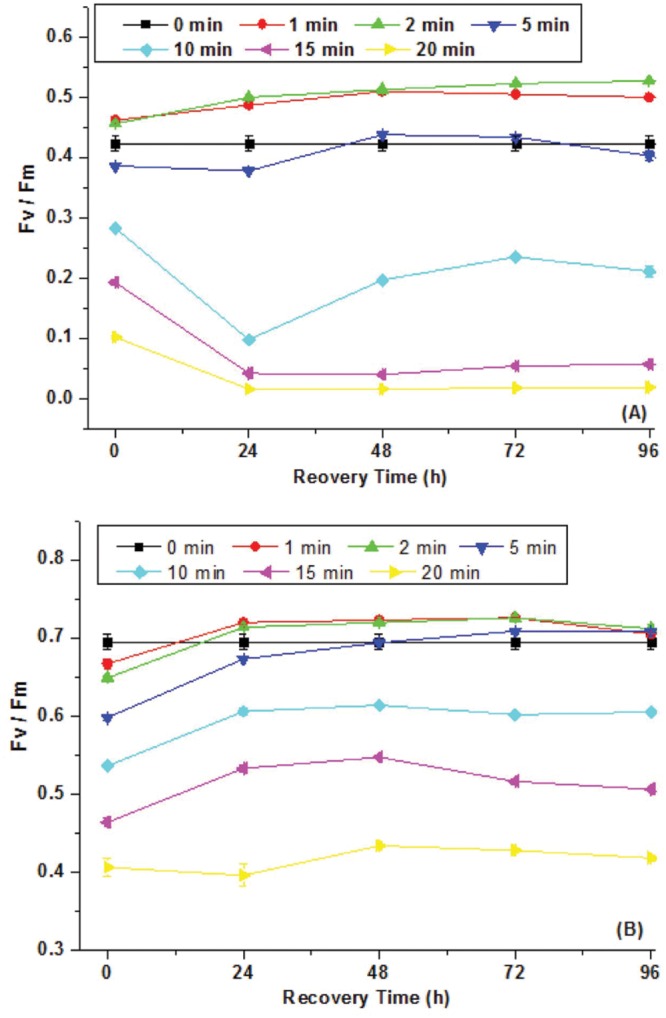
**The maximum photochemical efficiency of PSII, Fv/Fm (*N* = 3, ±SD), of *Synechocystis* sp. **(A)** and *Chroococcus minutus***(B)** cells exposed to UVBR for various time and its recovery curves up to 96 h**.

**Figure 1** showed Fv/Fm of *C. minutus* and *Synechocystis* sp. as a function of UVBR time. It was found that 1 and 2 min of UVBR increased Fv/Fm of *Synechocystis* sp. compared with the control. However, longer UVBR time reduced Fv/Fm and the reduction increased with radiation time. In the case of *C. minutus*, Fv/Fm was reduced under all periods of UVBR in comparison with the control and its reduction correlated with radiation time. After radiation treatments, the recoverability of growth activity of both cyanobacteria species was assessed by continuous determination on Fv/Fm for 4 days. It was interesting that Fv/Fm increased to higher levels than their controls for both *Synechocystis* sp. and *C. minutus* irradiated with UV-B for 1 and 2 min, suggesting that short term UVBR can improve their PSII activities. In the case of *Synechocystis* sp. Fv/Fm decreased from 0.43 to 0.1 irradiated after 10 min of UVBR and continued to decrease significantly during first recovering 24 h and then recovered gradually to about 0.2 at 48 h. In 15 and 20 min of UVBR treatments reduced Fv/Fm to 0.2 and 0.1, respectively. After removal of UVBR, Fv/Fm for these two treatments continued to decrease to near zero and no recovery was observed. This means that PSII was completely destroyed by 15 or 20 min of UVBR. For *C. minutus* exposed to UVBR for 10 min or longer, Fv/Fm recovered somewhat to values below the control (**Figure 1B**), indicating that PSII function was injured and could be recovered partly. These results showed that *C. minutus* was more tolerant to UVBR than *Synechocystis* sp. and the former had higher ability to recover from depression in Fv/Fm induced by long-term (5–20 min) UVBR than the latter.

A number of studies also showed that strong UVBR can damage PSII and cause a reduction in the maximal quantum yield (Fv/Fm; Karsten et al., 2007). Limited studies showed that PSII activity for UVBR stressed terrestrial and aquatic algal species showed contrasting recovery potential. Karsten et al. (2007) showed that Fv/Fm of three terrestrial algal species *Stichococcus* sp., *C. luteoviridis*, and *M. incise* and one aquatic algae *D. subspicatus* significantly declined under 3 days of radiation of 50 μmol photons m^-2^ s^-1^ PAR and 40 μW cm^-2^ UVB. Fv/Fm fully recovered for all the three terrestrial algal species while the aquatic algae recovered about 80% during12 days of recovery. Similarly, our study also indicates that the aquatic cyanobacterium is more susceptible to UVBR than the terrestrial species.

### Effect of UVBR on Cell Growth and EPS Production

**FIGURE 2:**
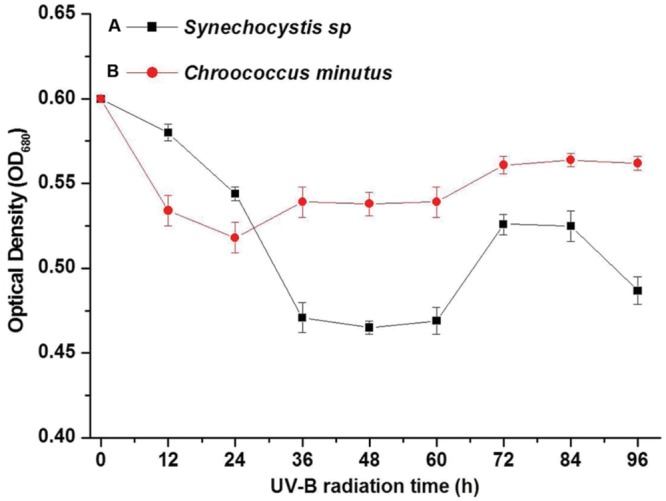
**The mean optical densities (OD_680_) (*N* = 3, ±SD) of *Synechocystis* sp. cells **(A)** and *C. minutus* cells **(B)** with UVBR time**.

The optical density of cell suspension at 680 nm (OD_680_) was used to indicate the living cell biomass. *Synechocystis* sp. and *C. minutus* showed different growth patterns during 4 days of continuous exposure to UV-B (**Figure 2**). The decreasing magnitude of the biomass of *Synechocystis* sp. was bigger than *C. minutus* during UVBR (**Figure 2**), implying that *C. minutus* was more tolerant to UVBR. Growth of *C. minutus* recovered at 24 h while *Synechocystis* sp. started its growth at 60 h, much later than *C. minutus*. This suggests that *C. minutus* can acclimate to UVBR more rapidly than *Synechocystis* sp.

During the treatments of UVBR and staining, no changes in cell shape were observed. When the EPS were stained with alcian blue, EPS exhibited a blue–green fluorescence. It was observed that most of EPS of *Synechocystis* sp. cells disappeared after UVBR (Supplementary Figures S1A,B). However, it seemed that UVBR increased production of EPS of *C. minutus* cells (Supplementary Figures S1C,D). The quantitative analysis confirmed the results of light microscopy. EPS yield of *Synechocystis* sp. decreased from 407.8 mg g^-1^ dry cell before UVBR to 70 mg g^-1^ dry cell after 12 h of UVBR whereas yield of EPS of *C. minutus* increased from 60.7 mg g^-1^ dry cell before UVBR to 198.2 mg g^-1^ dry cell after 12 h of exposure to UVBR. This indicates that EPS of *Synechocystis* sp. was decomposed under UVBR whilst production of EPS of *C. minutus* was stimulated by UVBR.

In previous studies, the cell growth received much more attention as it is considered to be an important physiological parameter that integrates all positive and negative stress effects in several biochemical processes within the cell (Karsten et al., 2007). The growth studies on macroalgae indicate that more cellular processes are impaired than just photosynthesis (Dring et al., 1996). The change of cellular metabolism could induce the secretion change of EPSs as it is regarded as one important response of microorganism to environmental stress. A few earlier studies reported responses of EPS of cyanobacteria to UV radiation. EPS production of *Microcoleus vaginatus* decreased significantly after radiation with UV-B (Chen et al., 2009). He and Häder’s (2002) study indicated that EPS of *Microcoleus vaginatus* had a significant protection against the oxidative stress and photosynthetic damage and thus reversed the negative effects of UV-B on survival. In previous studies, it has been observed similar result. For example, the production of extracellular glycan in *Nostoc commune* was stimulated threefold under UV-B and EPS had important effect on protection against UV-B (Ehling-Schulz et al., 1997).

### Effect of UVBR on Molecular Weight Distribution of EPS

**FIGURE 3:**
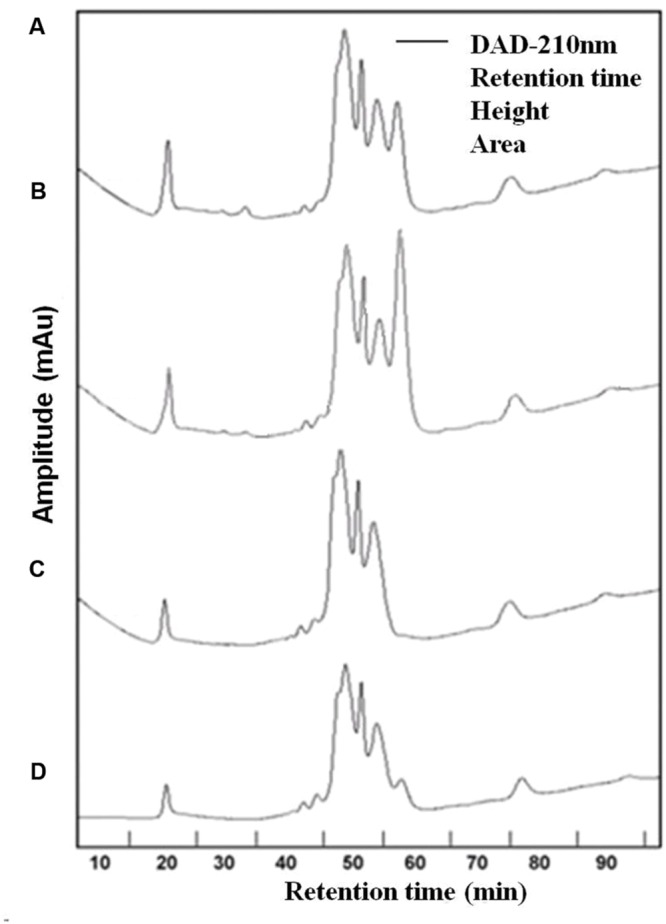
**High performance liquid chromatography (HPLC) chromatogram of EPS abstracted from *Synechocystis* sp. cells and *C. minutus* cells. (A)** EPS from *Synechocystis* sp.; **(B)** EPS from *Synechocystis* sp., was UV-B irradiated for 12 h; **(C)** EPS from *C. minutus*; **(D)** EPS from *C. minutus*, was UV-B irradiated for 12 h.

Three chromatographic fractions were separated from EPS of both cyanobacteria species untreated and treated with UVBR (**Figure 3**). Most of the molecular weights of both *Synechocystis* sp. and *C. minutus* fell into the range of retention time from 45 to 60 min. Two small peaks at 20 and 77 min were also observed for EPS of both cyanobacterial species. The area of peak at 20 min for EPS from *Synechocystis* sp. (**Figure 3A**) was a little bigger than that from *C. minutus* (**Figure 3C**), indicating the EPS of *Synechocystis* sp. contained more macromolecular substances than those of *C. minutus*. According to the organic carbon mode (Paul et al., 2012), the high-molecular weight matter with retention time less than 40 min was composed of mainly hydrophilic biopolymers, such as polysaccharides and proteins. The middle-molecular weight matter with retention time of 40–60 min could be humic acids, fulvic acids, and fractions of low-molecular weight acids. The minor peak at retention time >60 min represents the low-molecular weight neutral and amphiphilic compounds including sugars, alcohols and amino acids. The proportions of the substances in the three molecular weight ranges were shown in **Table 1**. EPS from *Synechocystis* sp. and *C. minutus* contained 11.5 and 7.7% of the MW > 94 kDa substances (mainly polysaccharides and proteins), respectively. After 12 h of UVBR, about 3.8 and 0.8% of the MW > 94 kDa substances in EPS from *Synechocystis* sp. and *C. minutus* were decomposed, respectively. Substances with MW in the range of 14.4–94 kDa (low-molecular weight acids) accounted for 62.9 and 53.1% in EPS from *Synechocystis* sp. and *C. minutus*, respectively. 9.8 and 3.6% of 14.4–94 kDa substances were decomposed due to 12 h of exposure to UV-B for *Synechocystis* sp. and *C. minutus*, respectively. In contrast to the decreases of proportion of substances with MW > 14 kDa, MW < 14 kDa substances (low-molecular weight neutral and amphiphilic compounds) increased from 25.5 and 20.2% before UVBR to 39.1 and 24.6% after 12 h of UVBR for *Synechocystis* sp. EPS and *C. minutus* EPS, respectively. The decreases of proportion of high MW substances and increases of low MW substances due to UVBR indicated that the high MW components were decomposed into low MW substances. 13.6% of MW > 14 kDa substances were decomposed into MW < 14 kDa molecules for *Synechocystis* sp. during UVBR, which was much more than the 4.4% for *C. minutus*, indicating that EPS of the aquatic *Synechocystis* sp. was more decomposable than the desert *C. minutus*.

**Table 1:** The molecular weight distribution of EPS from *Synechocystis* sp. and *Chroococcus minutus* before and after 12 h of UVBR.

Molecular weight(MW) range	Integral area (%)			
	SEPS	SEPS UV-B (12 h)	CEPS	CEPS UV-B (12 h)
MW > 94 kDa	11.5	7.7	6.9	6.1
14.4 kDa < MW < 94 kDa	62.9	53.1	72.9	69.3
MW < 14.4 kDa	25.5	39.1	20.2	24.6

The decomposition of EPS under UVBR is similar to the decomposition of dissolved organic carbon (DOC) caused by UV radiation. In a few earlier studies, effects of UV-radiation on DOC were evaluated using chromatographic techniques (Frimmel, 1998; Paul et al., 2012). These studies showed that transformation of DOC induced by UV radiation was characterized by reduction of macromolecular matter and an increase of low-molecular weight matter (Paul et al., 2012). Some studies also reported that UV radiation caused decomposition of large natural organic matter molecules to small ones (Lehtola et al., 2003).

### Effect of UVBR on Biochemical Characteristics of EPS

The pH, proteins content, and polysaccharides content of EPS from *Synechocystis* sp. and *C. minutus* during UVBR were listed in **Table 2**. The pH of *Synechocystis* sp. EPS was about 7 and pH of *C. minutus* was weakly acidic, 5.8. UVBR slightly increased pH of both EPS solutions. The change of pH might be attributed to the decomposition of EPS induced by UVBR (Stal and Krumbein, 1985). The TOC content showed contrary change patterns after exposure to UV-B. TOC content of *Synechocystis* sp. EPS decreased with increasing UVBR time, suggesting that EPS were decomposed due to UVBR. On the contrary, TOC content of *C. minutus* EPS showed an increasing trend after UVBR. This might be attributed to the incomplete oxidation of *C. minutus* EPS at 680°C (the combustion temperature of the TOC analyzer). With UVBR time, more EPS were decomposed into low MW components that could be more easily oxidized at 680°C by the TOC analyzer and this resulted in a small increase of TOC content. The contrasting response pattern of TOC content of *Synechocystis* sp. and *C. minutus* to UVBR suggests that EPS of *C. minutus* was more resistant to UV-B induced degradation than that of *Synechocystis* sp.

**Table 2:** Effects of UVBR on pH and composition of EPS from *Synechocystis* sp. and *C. minutus*.

	EPS	UVBR time				
		0 h	2 h	6 h	9 h	12 h
pH	*Synechocystis* sp.	6.98	7.03	7	7.15	7.12
	*C. minutus*	5.78	6.42	6.31	6.83	6.47
TOC (mg L^-1^)	*Synechocystis* sp.	2	1.7	1.7	1.4	1.5
	*C. minutus*	2	1.9	2.6	2.8	3
Protein (mg L^-1^)	*Synechocystis* sp.	0.110 ± 0.02	0.011 ± 0.005	0.003 ± 0.002	0.006 ± 0.006	0.010 ± 0.002
(*N* = 3, ±SD)	*C. minutus*	0.70 ± 0.02	0.59 ± 0.04	0.61 ± 0.08	0.46 ± 0.04	0.52 ± 0.08
Polysaccharide (mg L^-1^)	*Synechocystis* sp.	0.23 ± 0.007	0.08 ± 0.001	0.22 ± 0.006	0.15 ± 0.004	0.10 ± 0.001
(*N* = 3, ±SD)	*C. minutus*	0.11 ± 0.02	0.14 ± 0.03	0.17 ± 0.03	0.11 ± 0.02	0.19 ± 0.06

Extracellular polymeric substance of *Synechocystis* sp. had lower protein content and higher polysaccharides content compared to EPS of *C. minutus*. Protein concentration in EPS of *Synechocystis* sp. showed slightly fluctuating changes with increasing exposure time to UVBR, and finally decreased from 0.11 to 0.01 mg L^-1^ after 12 h of exposure to UVBR. Whereas protein concentration in EPS of *C. minutus* decreased significantly after UVBR and it had a good linear relation with the exposure time (*r*^2^ = 0.8133, *p* < 0.05). The decreasing of protein content in EPS after UVBR exposure indicated protein could be degraded by UVBR. The polysaccharides content in EPS of *Synechocystis* sp. decreased slightly after UVBR but that of *C. minutus* almost kept unchanged. This might be due to the differences in composition and structure of polysaccharides in EPS from contrasting environments. The degradation of proteins in EPS of both species could be attributed to ROS produced by UVBR (Kumar et al., 2004). The decomposition of EPS and the decreasing of protein content in EPS might explain the decreases of photosynthetic activity and biomass of *Synechocystis* sp. and *C. minutus* after UVBR.

### Effect of UVBR on EEM Spectra of SEPS and CEPS

**FIGURE 4:**
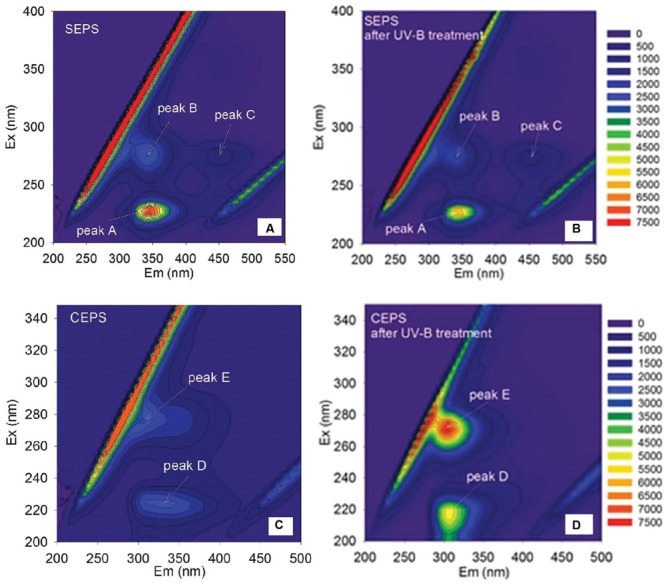
**Excitation emission matrix (EEM) spectra of *Synechocystis* sp. EPS before **(A)** and after **(B)** 12 h of UVBR, and of *C. minutus* EPS before **(C)** and after **(D)** 12 h of UVBR**.

The fluorescence spectra of EPS were shown in **Figure 4**. Two protein-like peaks (peak A and peak B) and one humic-like peak (peak C) were identified from EEM spectra of *Synechocystis* sp. EPS (**Figure 4A**). One protein-like peak (peak D) and one phenol-like peak (peak E) were identified from the EEM spectra of *C. minutus* EPS (**Figure 4C**). According to the peak position (**Table 3**), fluorescence peaks A and B could be assigned to secondary tryptophan-like fluorescence and tryptophan-like fluorescence, respectively (Song et al., 2010; Zhu et al., 2012).

**Table 3:** Comparison between the effects of UVBR on fluorescence properties of EPS from *Synechocystis* sp. EPS and *C. minutus* EPS, respectively.

UVBR time (h)	Ex(nm)/Em(nm) (Fluorescence Intensity)				
	*Synechocystis* sp. EPS			*C. minutus* EPS	
	Peak A	Peak B	Peak C	Peak D	Peak E
0	225/344 (7166)	275/342 (2528)	275/452 (1220)	225/326 (2693)	275/304 (3088)
12	225/342 (5961)	275/340 (2040)	275/452 (1143)	220/308 (5312)	270/304 (7524)

After 12 h of UVBR, the position of fluorescence peaks for *Synechocystis* sp. EPS changed little but the fluorescence intensity at the protein-like peak was reduced (**Figures 4A,B** and **Table 3**), indicating that some protein-like substances were degraded by UVBR. This is consistent with the result of molecular weight, indicating the proteins in *Synechocystis* sp. EPS were decomposed and this significantly reduced the proportion of the high molecular weight components. UV photodegradation favored larger molecules in NOM fragmentation (Frimmel, 1998). Some previous studies also show that photobleaching is due to the photodegradation of DOM and is often accompanied by a decrease in the molecular weight (Tremblay et al., 2007).

However, the position and intensity of fluorescence peak, especially protein-like peak, in spectra of CEPS changed significantly after UVBR (**Figures 4C,D** and **Table 3**). It was observed that there was one slight red shift of protein-like fluorescence peaks and fluorescence intensity at both protein-like peak and phenol-like peak increased significantly. This might be attributed to the change of structure and composition of fluorescence substances induced by UVBR, such as unfolds of some rigid fluorescent substances (Stal and Krumbein, 1985).

Extracellular polymeric substance play a key role in buffering against the adverse effects exerted by the environmental stressors such as UVBR because EPS physically serve as a barrier between the cell and the environment. However, the buffering capacity of EPS seems to be closely related to the habitat factors of the cyanobacteria live in. In the present study, the PSII activity of the desert species *C. minutus* showed much higher tolerance to UVBR than the aquatic *Synechocystis* sp., which can be explained by the different resistance of their EPS to UVBR. *C. minutus* inhabits the desert and it has been always exposed to high UV radiation while *Synechocystis* sp. lives in the surface water with less UV radiation. The long-term exposure to UV radiation results in more stable EPS that can effectively mitigate the adverse effects of UV radiation.

## Conclusion

The desert cyanobacterium species *C. minutus* showed higher tolerance of PSII to UVBR than the aquatic *Synechocystis* sp., and the inhibited PSII activity of *C. minutus* could be fully recovered while that of *Synechocystis* sp. could be partly recovered. Proteins in EPS of desert and aquatic species were significantly decomposed under UVBR, with the latter being more easily decomposed. The polysaccharides were much more resistant to UVBR than the proteins for both species. Polysaccharides of *Synechocystis* sp. were degraded slightly but almost no *C. minutus* polysaccharides were decomposed. The higher tolerance to UVBR of the desert cyanobacterium can be partly attributed to the higher resistance of its EPS to photodegradation induced by UVBR in comparison with the aquatic species.

## Author Contributions

WJS is the first author responsible for doing experiments and writing. XLP is the corresponding author. CXZ, DYZ, and SYM are coauthors.

## Conflict of Interest Statement

The authors declare that the research was conducted in the absence of any commercial or financial relationships that could be construed as a potential conflict of interest.
