# Supplementary material for: Different Resistance to UV-B Radiation of Extracellular Polymeric Substances of Two Cyanobacteria from Contrasting Habitats
Source: Front Microbiol. 2016 Aug 22;7:1208. doi: 10.3389/fmicb.2016.01208 (PMC4992692; doi:10.3389/fmicb.2016.01208)
Supplement: Supplementary file 1 [file Image_1.PDF]

Supplementary Materials

Different resistance to UVBR of extracellular polymeric substances of two cyanobacteria from contrasting habitats

Wenjuan Song[1], Chenxi Zhao[2], Daoyong Zhang[1,3], Shuyong Mu[1], Xiangliang Pan[1]*

[1]Xinjiang Key Laboratory of Environmental Pollution and Bioremediation, Xinjiang Institute of Ecology and Geography, Chinese Academy of Sciences, Urumqi 830011, China

[2]Xinjiang Academy of Environmental Protection Sciences, Urumqi, Xinjiang 830011, China

[3]State Key Laboratory of Environmental Geochemistry, Institute of Geochemistry, Chinese Academy of Sciences, Guiyang 550002, China

* Corresponding author: xlpan@ms.xjb.ac.cn (PXL) Tel: +86-991-7823156, Fax: +86-991-7823156

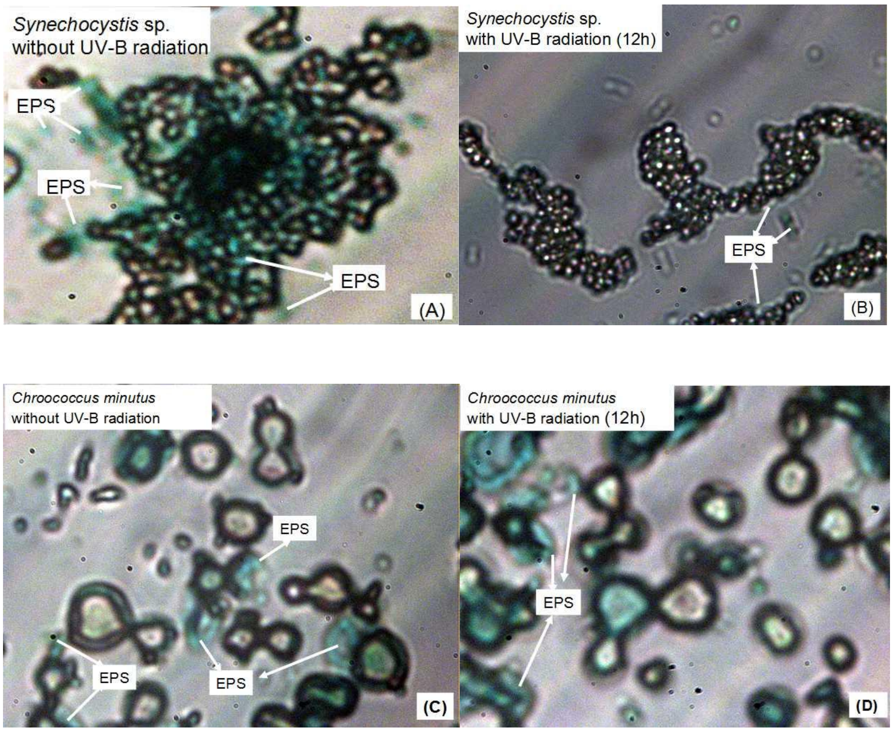

SFIGURE 1. EPS of *Synechocystis* sp cells (A) and *Chroococcus minutus* cells (C) before UVBR; and EPS of *Synechocystis* sp cells (B) and *Chroococcus minutus* cells (D) after 12h of UVBR .The blue-green region in images indicated the alcian blue stained EPS.
